# Cholangioscopy-targeted, ultrasound-monitored needle puncture for complete bilioenteric anastomotic occlusion in a 1-year-old liver transplant recipient

**DOI:** 10.1055/a-2961-1567

**Published:** 2026-09-30

**Authors:** Rui Chen, Jingyi Zhang, Tianhao Chen, Jie Zhang, Rongxing Zhou

**Affiliations:** 1 Department of Ultrasound 34753 West China Hospital of Sichuan UniversityChengduSichuanChina; 2 Division of Biliary Surgery, Department of General Surgery 34753 West China Hospital of Sichuan UniversityChengduSichuanChina

Biliary strictures follow 4 to 12% of pediatric liver transplantations within 2 years. 1​ A 1-year-old boy who had undergone liver transplantation with Roux-en-Y hepaticojejunostomy for cholestatic cirrhosis presented 5 months later with recurrent jaundice. Magnetic resonance cholangiopancreatography showed a severe bilioenteric anastomotic stricture ( Fig. 1a ), and the guidewire failed to cross the anastomosis during two successive percutaneous transhepatic biliary drainage (PTCD) procedures ( Fig. 1b, c ), indicating complete occlusion. Given the infant’s age, the morbidity of surgical revision, and the need to limit ionizing radiation, percutaneous transhepatic cholangioscopy (PTCS) was chosen for in situ recanalization. 2​

**Fig. 1:**
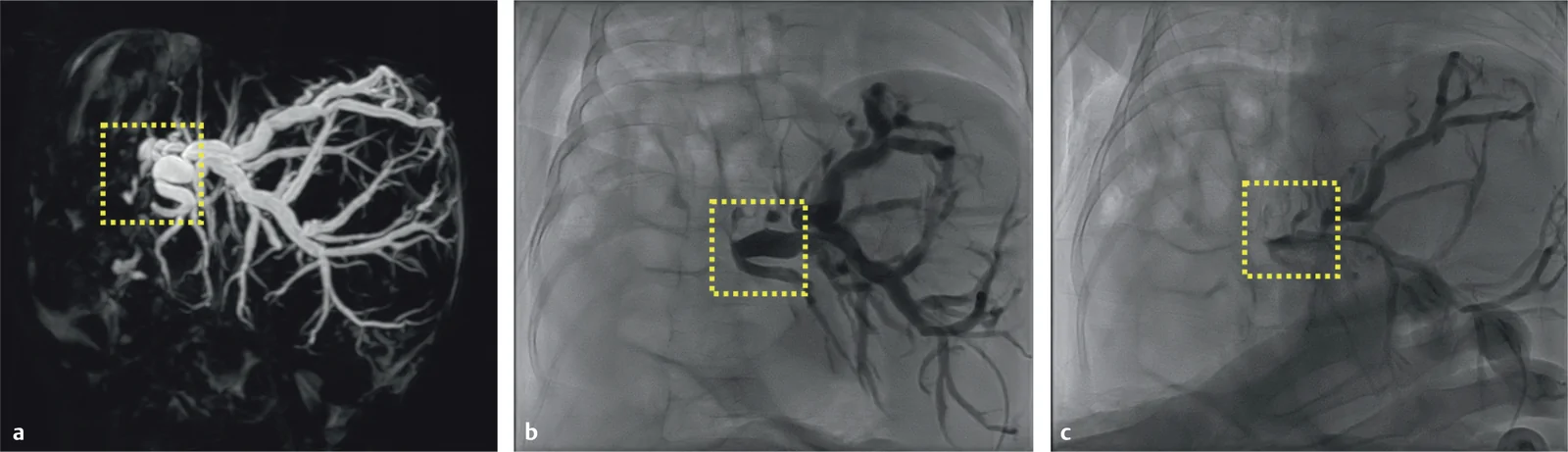
Preoperative imaging assessment. ( **a** ) Magnetic resonance cholangiopancreatography showing intrahepatic bile duct dilatation with a severe bilioenteric anastomotic stricture. ( **b** and **c** ) During both percutaneous transhepatic biliary drainage (PTCD) procedures, the guidewire failed to traverse the strictured segment of the anastomosis.

A 16-Fr working sheath was advanced along the mature PTCD tract. Rigid cholangioscopy with intraoperative cholangiography confirmed a blind-ending, completely occluded anastomosis ( Fig. 2a, b ), and repeated guidewire probing identified no residual channel ( Fig. 2c ), so direct puncture of the scar plate was undertaken ( Video 1 ). Under direct vision, the cholangioscopy defined the entry point and the puncture axis ( Fig. 3a ), while transabdominal ultrasound simultaneously displayed the needle trajectory, the afferent jejunal limb, and the adjacent hilar vessels in real time ( Fig. 3b ). The plate was traversed at the first attempt, without vascular injury. The newly created tract was sequentially dilated over a guidewire with 8-Fr and 10-Fr dilators ( Fig. 4a ); cholangiography confirmed unimpeded contrast flow into the jejunum without leakage ( Fig. 4b ), and a 16-Fr biliary catheter was left across the recanalized anastomosis.

**Fig. 2:**
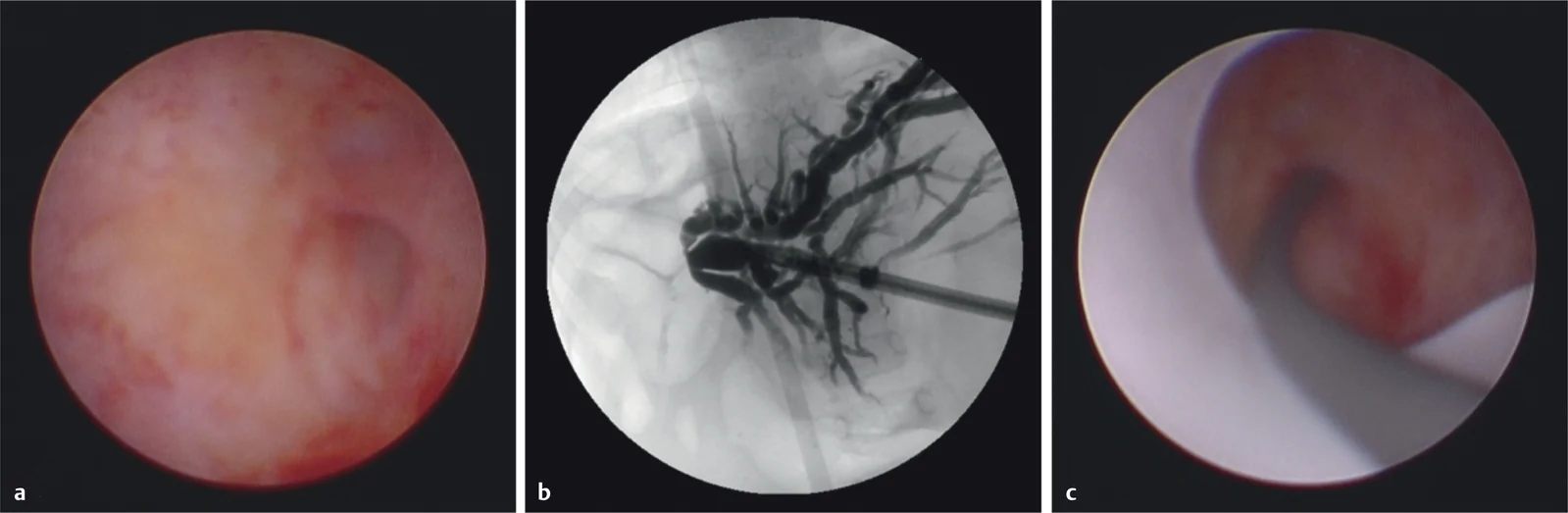
Rigid cholangioscopy combined with cholangiography confirming complete anastomotic occlusion and failed guidewire probing. ( **a** ) A direct cholangioscopic view showing the bilioenteric anastomosis to be completely occluded. ( **b** ) Intraoperative cholangiography showing contrast filling the intrahepatic bile ducts and terminating abruptly at the anastomotic blind end, with no passage of contrast beyond it. ( **c** ) A cholangioscopic view showing the conventional guidewire reaching the occluded blind end without finding any channel.

**Video 1** A completely occluded bilioenteric anastomosis was recanalized in situ by needle puncture under direct cholangioscopic vision with real-time ultrasound guidance in a 1-year-old liver transplant recipient.


**Fig. 3:**
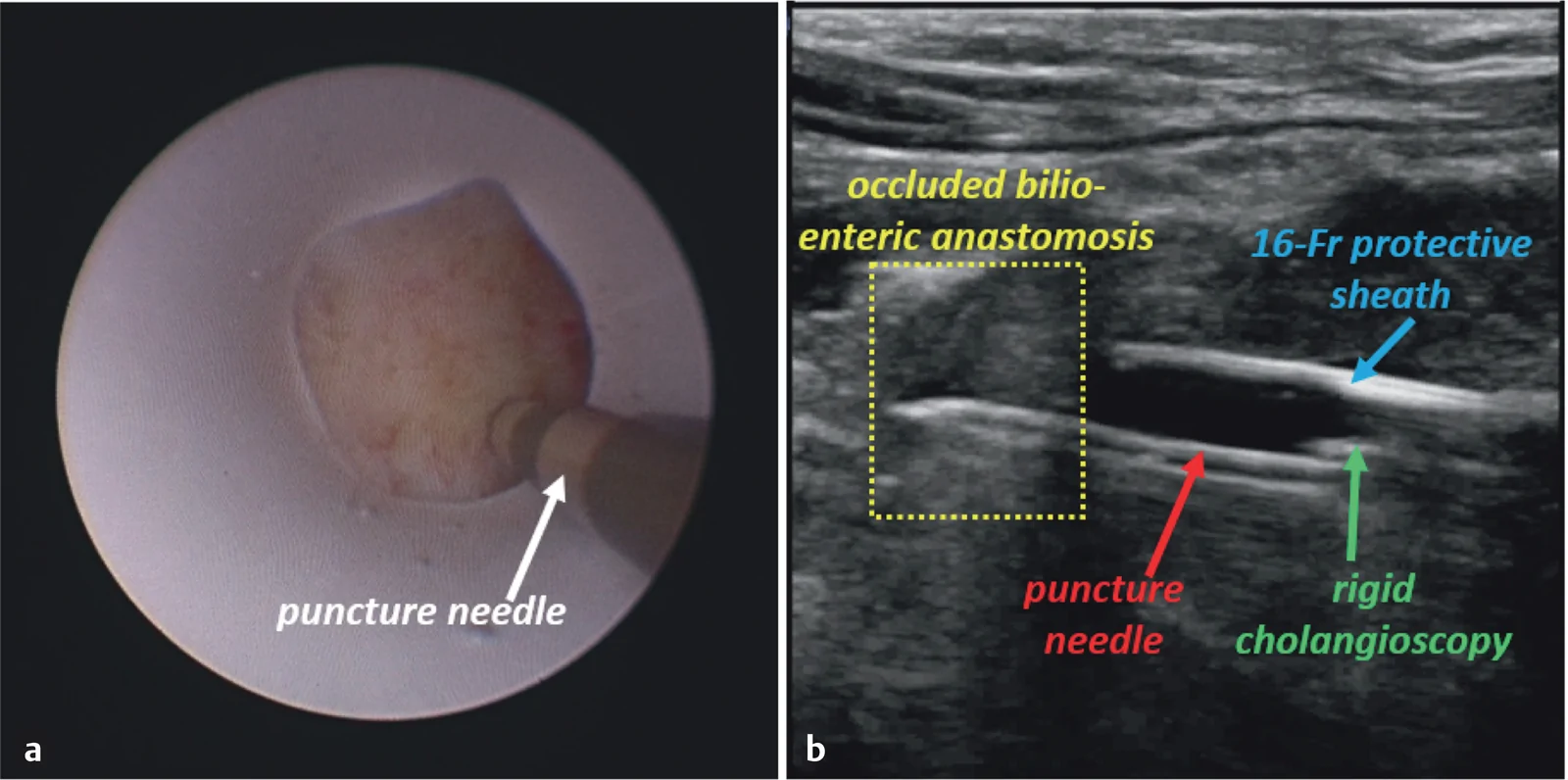
Dual-modality puncture of the occluded bilioenteric anastomosis under direct cholangioscopic vision ( **a** ) with real-time ultrasound guidance ( **b** ).

**Fig. 4:**
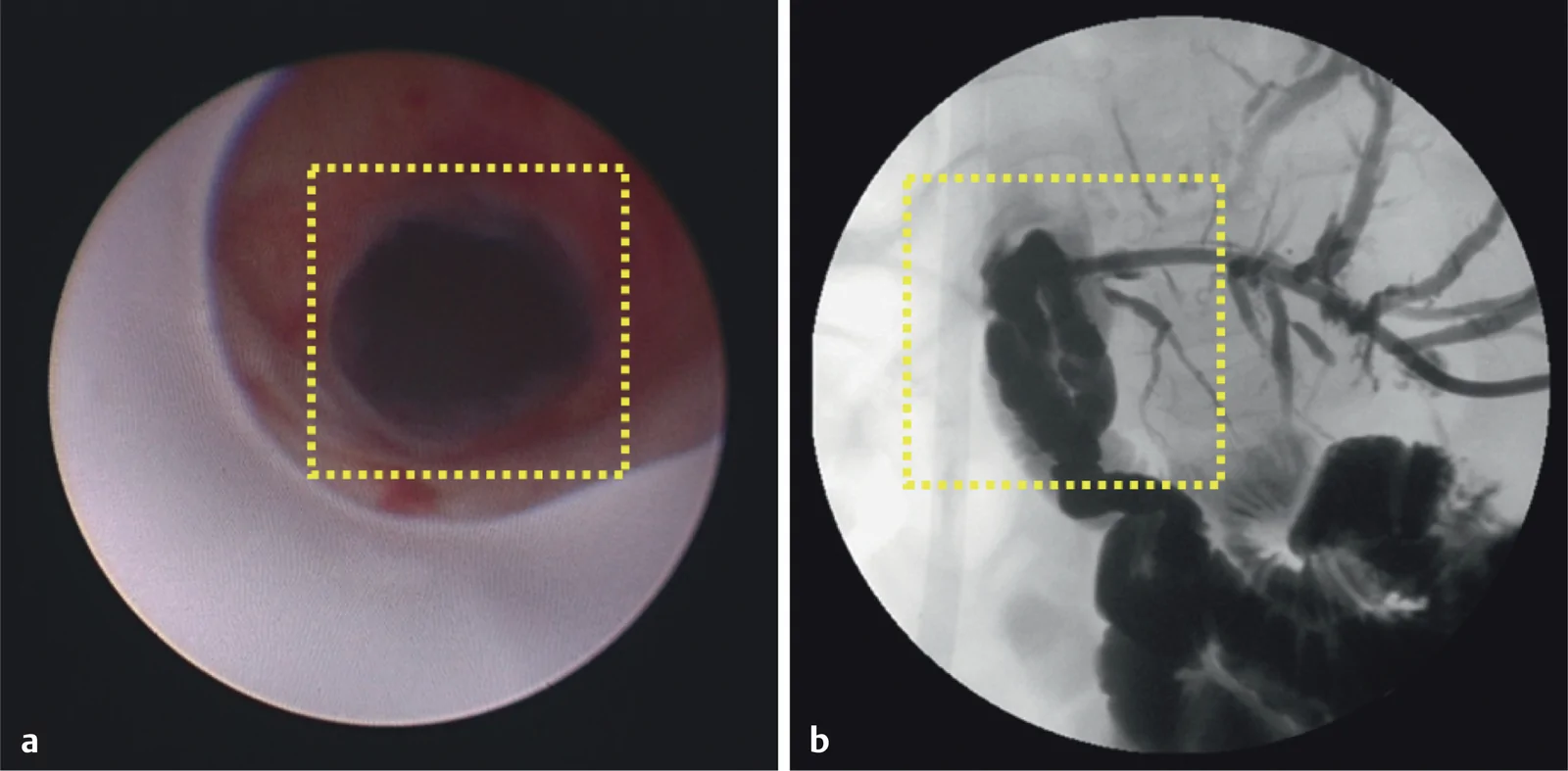
Sequential dilation of the puncture tract at the occluded bilioenteric anastomosis, with cholangiographic confirmation. ( **a** ) A direct cholangioscopic view after recanalization, showing a widely patent anastomosis with no bile leakage or bleeding. ( **b** ) Cholangiography after dilation, showing contrast passing through the recanalized bilioenteric anastomosis into the bowel lumen, without bile leakage.

No bleeding, bile leak, or bowel perforation occurred. Jaundice resolved rapidly and liver function normalized ( Fig. 5a ). After 6 months, the anastomosis remained widely patent on cholangiography ( Fig. 5b ) and the catheter was removed.

**Fig. 5:**
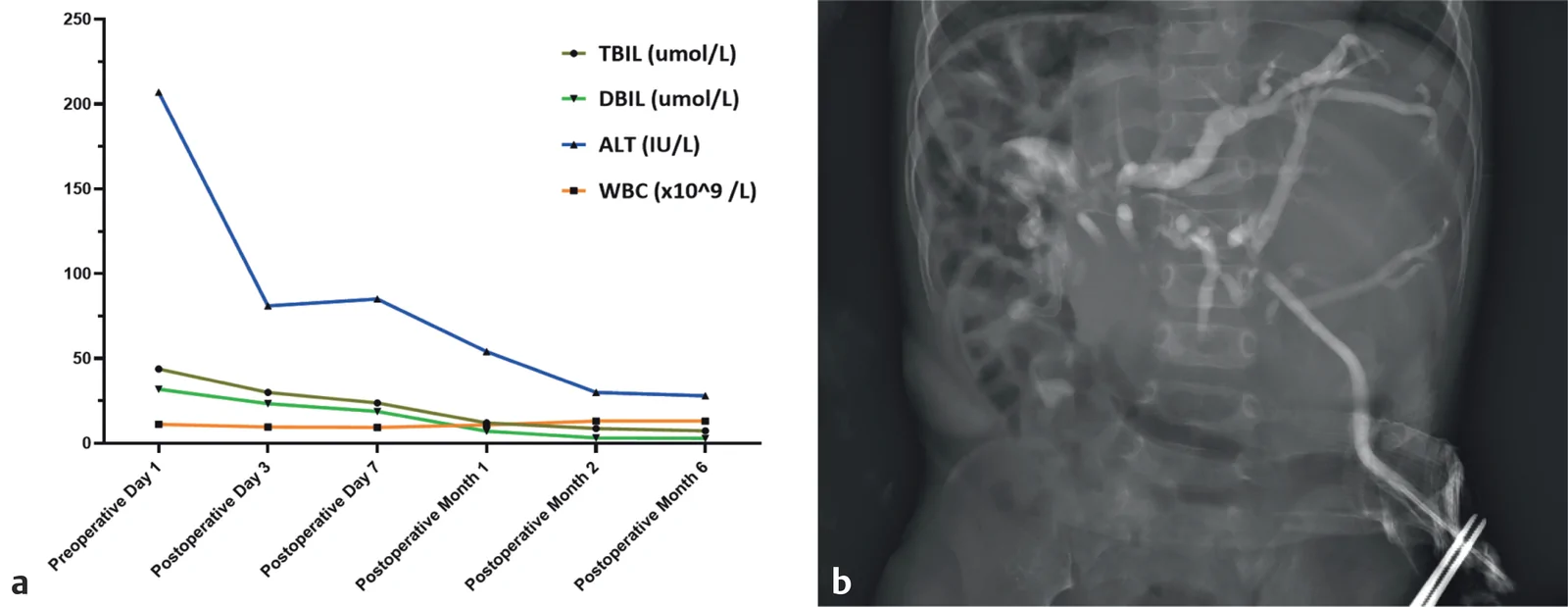
Liver function during follow-up and cholangiography after 6 months. ( **a** ) Trends in liver function parameters during the postoperative follow-up. ( **b** ) Cholangiography through the drainage catheter at 6 months, showing contrast passing freely through the recanalized tract into the jejunum, with no stricture or obstruction at the bilioenteric anastomosis.

Cholangioscopy resolves the target that fluoroscopy and ultrasound cannot define at a fibrotic blind end, while ultrasound supplies the depth and vascular information the cholangioscopy cannot see. Their combination extends PTCS recanalization from strictures that retain a traversable lumen to occlusions in which no residual orifice exists, in a patient younger than any previously reported. 3​ 4​ ​5​

Endoscopy_UCTN_Code_TTT_1AR_2AL

## Declaration of GenAI Use

The authors confirm that no generative artificial intelligence was used in the preparation of this manuscript.
